# A Preliminary Look

**Published:** 1920-05-15

**Authors:** 


### THE ROYAL ACADEMY.

### I.—A Preliminary Look.

Even if there is a member of the medical profession who has no interest in the art of the day for its own sake, he cannot afford to ignore the summer exhibition at the Academy, for it affords one of the most useful mirrors that we have of the trend of thought of our nation from year to year.

On its walls we see reflected our conservatism and radicalism, the ways in which we stand still and the ways in which we change. The Academy is a great British institution, and as such exemplifies our most striking characteristic compromise. We cling to our old lines of thought, and view all new ones at first with disfavour; then the new lines of thought thrust themselves upon us, and we are bound to notice them; but by this time they have grown older and thrown off some of the exuberant crudities of youth, so that when they are admitted to the fold of orthodoxy they have acquired a moderation with which our conservative ideas can compromise. That this method has its disadvantages we are constantly being told by every advanced member of any new school of thought. That it has its advantages is less seldom trumpeted about, but is more completely true, for by it certain freak schools are weeded out, and those which have stability are able to combine with what is good in the old, and not spring up to exuberant isolated growth. And so the course of progress, though it may be somewhat slow, is steady and kept in a reasonably straight path.

### A Readjustment.

The moment that we enter the Exhibition we are faced with the fact that Room VI., which by tradition has been allotted to oil paintings, has this year been given over to sculpture. In this way the overcrowding in the Lecture Room which we observed last year has been avoided, and room has been found for an increased number of examples of this form of the Fine Arts. For this encouragement of sculpture we have nothing but praise; we feel that in the past we have been all too negligent of it. We have treated it rather as a glorified branch of the stonemason's craft rather than as an art unsurpassed by her sisters as a means of expression, and one the examples of which last so long that the ideals of to-day can be handed down by it to posterity in the way that that tornado of energy of 5,000 years ago, Thothmes the Great, looks down upon us in the Cairo Museum to-day. Nor is this increased interest in sculpture entirely governed by the demand for war memorials of all kinds arising from the war, for the most striking and most delightful aspect of the change is shown by the marked increase of portrait busts, not of stately gentlemen for the purpose of record in their clubs, their colleges, or their institutions, but of little children for the delight of their parents and their loved ones. Most of these are charming, but we would specially recommend the smile of little Ivy Shearer as depicted by Isobel Donaldson in No. 1,364.

### A Problem of To-day.

In No. 558, entitled "Pay or Quit," Anna Airy has tackled the problem of poverty as it is seen to-day. In a one-roomed tenement, sparsely furnished, the rent-collector sits, his bag on the floor, his fist banged down on a large ledger that lies upon the table. The tenant, who has sold his shirt, is restrained from attacking him by his wife, while the two children look on with interest and the furniture-removal man stands at the door with none. The painting is effectual, nay, striking. It adds a touch of realism and of the sordidness which, for some unknown reason, is apt to be associated with the real to one of those subject pictures which delighted our grandparents sixty to a hundred years ago; but we doubt very much whether our grandchildren will gather anything from it, except that the rent-collector of to-day is a very unpleasant sort of gentleman. No doubt there are such rent-collectors, since Miss Airy has painted one, but he differs much from the unassuming little men or the bonny pleasant-looking women with their very smart boots whom we see collecting the rents of a Monday morning in a certain part of S.E. London.

### A Contrast.

In No. 679 (The Shadow of Hunger), Blamire Young has approached the same subject in a very different way. The scene is the Victoria Bridge Gardens looking eastward. In a bright light are the Houses of Parliament with the gardens in the foreground. Across them the setting sun has thrown an enormous shadow from Rodin's "Burghers of Calais." The gaunt figures of the hungry men are thrown against the building, so that you feel those within cannot be heedless of its presence.

